# Defect‐Functionalization‐Mediated Tunneling Drives Nonlinear Photoemission in Perovskites

**DOI:** 10.1002/advs.75220

**Published:** 2026-04-09

**Authors:** Hang Ren, Xi Zhang, Yicheng Wang, Shuai Wang, Zhi Wang, Yumeng Li, Jikai Yang, Xin Wang, Weijun Chen, Yining Mu

**Affiliations:** ^1^ School of Physics Changchun University of Science and Technology Changchun China; ^2^ School of Computer Science and Technology Changchun University of Science and Technology Changchun China

**Keywords:** defect functionalization, defect‐mediated tunneling, nonlinear photoemission, perovskite photocathode, quantum efficiency multiplication

## Abstract

The deliberate functionalization of defects represents a largely unexplored frontier in advanced optoelectronics. Within Einstein's photoelectric framework, we elucidate how deep‐level defects in CsPbBr_3_ films act as functional mediators to govern carrier transport, interfacial electron escape, and quantum efficiency multiplication via a defect‐mediated tunneling process. First, in solid‐state photonics, it overcomes strong exciton binding in an Au/CsPbBr3/Au transistor, yielding a >70‐fold photoconductive gain at sub‐nA dark current and a spectral response extension of >0.88 eV. Second, in vacuum electronics, electron beam bombardment creates localized electropositive centers, forming a defect‐functionalized activation layer that induces pronounced band bending and shifts interface dominance from luminescence to photoemission, offering a robust alternative to chemical methods. Thirdly, we demonstrate transient quantum efficiency multiplication that reaches 16.8% under 266 nm pulsed excitation and achieves a 34 dB gain at 355 nm, functionally establishing a photoemissive comparator. By transforming the conventional role of defects from detrimental to functional, this work represents “defect‐functionalized optoelectronics” as an alternative design route for advanced nonlinear device function.

## Graphical abstract

This cover illustration depicts CsPbBr_3_ perovskite nanocrystals embedded with functionalized defect vacancies, where electrons migrate through defect‐mediated pathways and emit as lightning‐like beams. The imagery visualizes the core discovery of our work—defect‐functionalization‐mediated tunneling enabling nonlinear photoemission, where deep‐level defects transform from recombination centers into dynamic conduits for efficient electron escape and quantum efficiency multiplication.

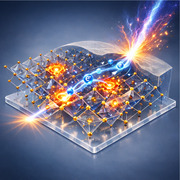

## 1 Introduction

One of the most important and technologically relevant phenomena in light‐matter interaction is the photoelectric effect [1, 2, 3], whereby photons eject electrons from matter. State‐of‐the‐art photocathodes can be categorized into three primary types. The first type comprises metallic photocathodes (e.g., Mg, Au) [4, 5]. The second type includes multi‐alkali photocathodes (e.g., Cs_2_Te, K_2_CsSb) [6, 7]. The third type involves III‐V semiconductor photocathodes (e.g., GaAs, GaN) [8, 9]. Non‐metallic photocathodes leverage negative electron affinity (NEA) induced by surface dipoles that lower the vacuum level (E_vac_) below the conduction band minimum (CBM). This enables high quantum efficiency (QE) spanning ultraviolet (UV) to infrared wavelengths [10, 11], surpassing metallic photocathodes by several orders of magnitude [12, 13]. Via chemical activation techniques such as Cs‐deposition [14], photo‐excited electrons can efficiently spill over the lowered interface barrier, enabling high QE exceeding 20% in linear photoemission [15]. High‐efficiency linear photoemission advances the exploration of novel physical phenomena and key technologies such as image intensifiers for night imaging [16, 17], electron microscopy [18, 19], free‐electron lasers [20, 21], and photomultipliers [22]. While diverse photoemission mechanisms and advanced photocathodes have been proposed in prior studies [23, 24], a nonlinear photoemission theory enabling transient QE multiplication—functionally analogous to a photoemissive comparator—remains unrealized. Establishing such a nonlinear photoemission framework would critically promote ultrafast optoelectronics and super‐resolution imaging technologies, such as THz emission sources [25, 26] and next‐generation lithography [27, 28]. As a type of versatile and solution‐processable semiconductor, metal halide perovskites exhibit exceptional properties, such as carrier mobility exceeding traditional quantum dots by two orders of magnitude [29, 30]. Moreover, they exhibit pronounced nonlinear optical responses, including strong third‐order nonlinear susceptibility [31] and large two‐photon absorption cross‐sections [32]. Indeed, prior studies have established CsPbBr_3_ as an excellent nonlinear emitter, as evidenced by its transient luminescence under near‐infrared ultrafast excitation [33, 34]. Furthermore, unlike traditional III‐V semiconductors, perovskite quantum dot films exhibit significant defect tolerance at practical device scales. Even with lattice defect densities exceeding 20%, the luminescence intensity of CsPbBr_3_ films remains largely uncompromised [35]. This remarkable defect tolerance suggests a potential to harness defects, rather than be limited by them. Critically, however, CsPbBr_3_ exhibits positive electron affinity (PEA), contrasting sharply with the NEA III‐V photocathodes. This PEA characteristic imposes fundamental energy barriers: the bulk ionization energy (IE, energy difference from valence band maximum (VBM) to E_vac_) ranges from 5.65 ± 0.1 eV [36, 37], while its work function (W_f_, energy difference from Fermi level (E_f_) to E_vac_) lies 4.7 ± 0.2 eV [38]. These barriers severely inhibit photo‐excited electron escape, limiting the external QE to ∼1% [39] under continuous wave (CW) ultraviolet‐C (UVC, ≤280 nm) excitation—one order of magnitude lower than NEA III‐V photocathodes [15]. This low CW‐QE baseline, however, establishes the necessary headroom for substantial QE multiplication under transient pulsed excitation. Defect engineering has enabled notable commercial advances, and recent studies have begun to harness defect‐assisted transport and recombination in areas such as photocatalysis and ultrafast optoelectronics [40, 41, 42, 43, 44]. In this work, within Einstein's photoelectric framework, we here deliberately repurpose these deep‐level defects of CsPbBr_3_ films from detrimental traps into functional mediators to distinctly achieve QE multiplication and nonlinear photoemission. The photoemission process in a CsPbBr_3_ photocathode can be delineated into three critical steps. To address Step II (efficient charge transport), we employ electron beam (EB) bombardment to create a surface rich in deep‐level defects in an Au/CsPbBr_3_/Au transistor, establishing a defect‐mediated tunneling mechanism that enables >70‐fold photoconductive gain—thereby overcoming the charge‐transport bottleneck. For Step III (interfacial electron escape), we introduce a defect‐mediated activation mechanism via EB bombardment, whereby induced deep‐level defects aggregate to form localized electropositive centers at the surface/vacuum interface. These centers reconfigure interfacial functionality by shifting dominance from luminescence to photoemission, enabling efficient escape of low‐energy electrons. Most significantly, we identify defect‐mediated tunneling during secondary excitation in Step I as the physical origin of nonlinear photoemission, which enables transient QE multiplication in CsPbBr_3_ photocathodes, demonstrating a peak transient QE of 16.8% at 266 nm with >34 dB gain under 355 nm pulsed excitation.

## 2 Defect‐Mediated Tunneling Overcomes Exciton Binding for Carrier Transport

Unlike CsPbBr_3_ photovoltaic devices [45, 46], where electron and hole transport layers (ETL/HTL) facilitate free carrier separation, the substantial exciton binding energy (E_b_ ≈ 40 meV) in CsPbBr_3_ [47, 48, 49] severely limits exciton dissociation and photo‐excited electron transport in the photocathode. This E_b_ exceeds the room‐temperature thermal energy (k_BT_ ≈ 25 meV) by a factor of 1.6, presaging that pristine films maintain efficient photoluminescence (PL) while excitons remain bound rather than dissociating into free carriers. To directly probe this transport limitation and address it via defect functionalization, we fabricated an Au/CsPbBr_3_/Au (metal/semiconductor/metal, MSM) transistor where focused EB bombardment (within a scanning electron microscope, SEM) was deliberately used to engineer deep‐level defects into functional mediators for carrier transport. As shown in Figure 1a, a hexane‐diluted CsPbBr_3_ solution was spin‐coated onto interdigitated Au electrodes. The pristine CsPbBr_3_ film exhibits a bandgap of 2.42 eV, with a dominant low‐temperature PL peak at 513 nm, as presented in the inset of Figure 1a. Additional peaks within 0.11 eV of the band edge are attributed to shallow‐level defects (E_s_). Using established references and ultraviolet photoelectron spectroscopy (UPS) testing results of Figure 1a, the E_f_ of CsPbBr_3_ is theoretically positioned ∼1 eV above the VBM, calculated as IE (5.65 ± 0.1 eV) [36] minus W_f_ (4.7±0.2 eV) [38], yielding a theoretical electron affinity (χ≈ IE—E_g_ = 3.23 ± 0.1 eV). Although the contact between Au (work function, W_Au_ = 5.1 eV) [50] and CsPbBr_3_ (W_f_, 4.7 ± 0.2 eV [38]) is theoretically expected to form an Ohmic contact for holes, our solution‐processed MSM transistor exhibits negligible dark current (I_dark_). We attribute this to a high density of deep‐level defects introduced at the Au/CsPbBr_3_ interface during spin‐coating, effectively creating a heavily doped interface. This induces significant downward band bending and elevates local work function at the CsPbBr_3_ interface. The resulting high density of interfacial states triggers pronounced Fermi‐level pinning, directly evidenced by the minimal I_dark_ even under high bias voltages (U_Au_). This phenomenon is widely reported in metal contacts to solution‐processed perovskite films [51, 52]. Notably, photocurrent (I_p_) also remains minimal even under increasing bias voltage (U_Au_), as excitons remain bound and cannot dissociate into free carriers. Specifically, in CsPbBr_3_ films with near‐ideal photoluminescence quantum yield (PLQY), photo‐excited electrons confront severely restricted carrier transport in Step II. This bottleneck leads to extremely low external QE in photoemission, as it prevents photo‐excited electrons from reaching the surface. However, the in situ “transmission‐mode” energy‐dispersive X‐ray spectroscopy (EDS) characterization of the MSM transistor, integrated within the SEM system of Figure 1b, directly revealed a pronounced transformation in its photoelectric properties during focused EB bombardment. Critically, the “transmission‐mode” EDS mapping of Figure 1c (pre‐ vs. post‐EB bombardment) revealed that high‐energy EB bombardment deliberately induced spatially heterogeneous bromine loss in the biased (U_Au_ = 50 V) channel—this was designed to functionalize bromide vacancy‐related deep‐level defects. Consistent with this, the low‐temperature PL spectrum in Figure 1c showed an enhanced near‐infrared (NIR) emission band (1025–1075 nm), which confirms these bromide vacancy defects (E_t_ = 1.18 ± 0.03 eV above VBM) are functionalized to mediate exciton dissociation and free‐carrier generation. This attribution is supported by the complementary EDS mapping, which revealed a spatially heterogeneous loss of bromine, confirming that EB bombardment introduced these deep‐level defects into the CsPbBr_3_ surface or upper layers. The observed NIR emission (1025–1075 nm) maps to defect levels at E_t_ = 1.18 ± 0.03 eV above VBM, defects capable of mediating exciton dissociation and free carrier generation. The energy‐band diagrams of Figure 1d schematically illustrate carrier transport models via defect‐mediated tunneling. The spatial variation of the external electric field, derived from Poisson's equation under the depletion approximation, creates a position‐dependent electric field distribution ε(x, U_Au_) near the electrode interface. This ε(x, U_Au_) tilts the energy landscape of the MSM transistor, effectively thinning the barrier between the conduction band (E_c_)/valence band (E_v_) and the defect state (E_t_). This barrier reduction, termed the Equivalent Barrier Reduction (EBR) and denoted Δϕ_bending_(x, U_Au_), is quantified by integrating the electric field over a characteristic tunneling length λ (from x to x+λ). The λ is a characteristic length scale for tunneling. The electric field distribution ε(x, U_Au_) depends on the bias voltage, transitioning from a triangular profile at unsaturated bias to a trapezoidal one at saturation voltage (U_sat_). The explicit expression for Δϕ_bending_(x, U_Au_) is derived as Equation (1).

(1) $$\begin{array}{ccc} & & \Delta \phi_{bending}\left(x,U_{Au}\right)\\ & & \;=\int_{x}^{x+\lambda }\varepsilon \left(x^{\prime },U_{Au}\right)dx^{\prime }\\ & & \;=\left\{\;\begin{array}{c} \frac{2\lambda U_{Au}}{w_{d}}\left(1-\frac{x}{w_{d}}\right)\;if\;\;U_{Au}<\;U_{Sat\;}\;\\ \;\lambda \cdot \varepsilon_{max}\;if\;\;U_{Au}>\;U_{Sat}\;and\;x\leq x_{0}\;\\ \lambda \cdot \varepsilon_{max}\left(1-\frac{x-x_{0}}{\left(w_{d}^{\prime }-x_{0}\right)}\right)\;if\;U_{Au}>\;U_{Sat}\;and\;x>x_{0} \end{array}\right.\; \end{array}$$

where w_d_/w_d_’ are the depletion widths under triangular/trapezoidal interfacial electric fields. The ε_max_ is the interfacial field that reaches its maximum value. The value at the interface (x = 0) is particularly important and described as 2λU_Au_/w_d_ in that triangular model or λ·ε_max_ in that trapezoidal model. The total dark current is then obtained by integrating the local tunneling contribution throughout the depletion region (w_d_ or w_d_’). The local tunneling probability from E_v_ to E_t_, and hence the dark current density (J_dark_), is exponentially sensitive to the EBR. Its expression can be described as Equation (2) by the Wentzel‐Kramers‐Brillouin (WKB) approximation.

(2) $$\begin{array}{ccc} & & J_{dark}\propto P_{tun}^{E_{v}->E_{t}}\propto \int_{0}^{w_{d}/w_{d}^{\prime }}exp\\ & & \;\times \left\{-\frac{2\sqrt{2m_{e}^{*}}\lambda }{\hbar }{\left[\left(E_{t}-E_{v}\right)-\Delta \phi_{bending}\left(x,U_{Au}\right)\right]}^{1/2}\right\}dx \end{array}$$

where ℏ is the reduced Planck constant, and m_e_* is the electron effective mass on top of E_v_. The generation process of this defect‐mediated dark current is depicted in Figure 1d. Under higher reverse bias (U_Au_), steeper band bending non‐linearly promotes the tunneling of valence‐band electrons into the defect states (E_t_), generating free holes in the E_v_. Prior to the relaxation (characterized by time τ_r_) of these tunneling electrons within the defect states, the electric field triggers the transport of the generated free holes, contributing to the dark current. This results in a free‐hole‐dominated gain expressed as G_h_ = τ_r_/τ_h_, where τ_h_ is the hole transit time across the channel in Figure 1d. Specifically, the reverse electric field is strongly confined within the high‐field depletion zone (0≤x≤w_d_), with field strength decaying sharply beyond this zone. Consequently, in the non‐depletion regions (x>w_d_), the absence of a strong field leads to slow diffusive transport, severely impeding hole current accumulation. This bottleneck effect is the fundamental reason why the overall dark current remains clamped at sub‐nA levels, as observed in Figure 1e. The sub‐nA dark current also corroborates the spatially confined nature of the tunneling process itself. To further investigate the chemical origin of the defects, we performed X‐ray photoelectron spectroscopy (XPS) analysis on the CsPbBr_3_ film before and after EB bombardment. As shown in the inset of Figure 1e, a significant decrease in the Br 3d signal intensity is observed after bombardment, indicating bromine depletion in the near‐surface region. This marked change in the interfacial chemical environment is consistent with the formation of bromine‐vacancy‐related defects, which are widely reported as dominant deep‐level defects in halide perovskites [53, 54, 55, 56]. The XPS results in Figure 1e, the low‐temperature NIR PL spectrum, and the EDS mapping in Figure 1c collectively provide mutually reinforcing evidence.

FIGURE 1Carrier Transport of Defect‐mediated Tunneling in Au/CsPbBr_3_/Au Transistor. (a) Device model and detailed parameters of MSM Transistor. (b) In situ “transmission‐mode” EDS characterization accompanying photoelectric property transformation. (c) Characterization of low‐temperature PL spectrum and EDS mapping for CsPbBr_3_ film pre‐ and post‐EB bombardment. (d) Carrier dynamics model in the MSM transistor under different excitation conditions. (e) XPS characterization and I_dark_‐U_Au_ characteristics under defect‐mediated photoconduction. (f) I_p_‐U_Au_ evolution of defect‐mediated tunneling for wavelength‐dependent photoresponse. (g) Transient response and dynamic fingerprint of the MSM transistor under pulsed excitation.

**Figure:**
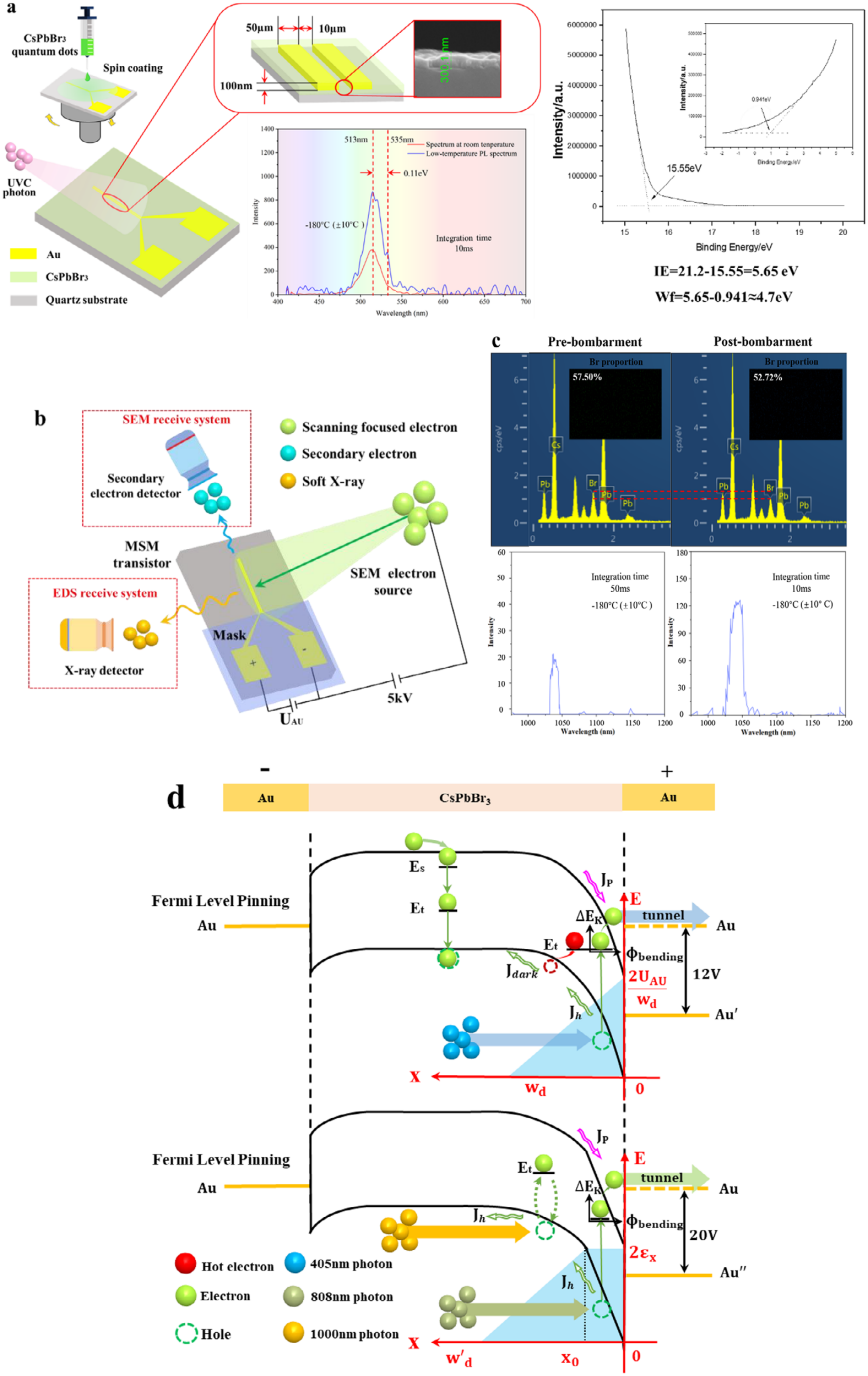

**Figure:**
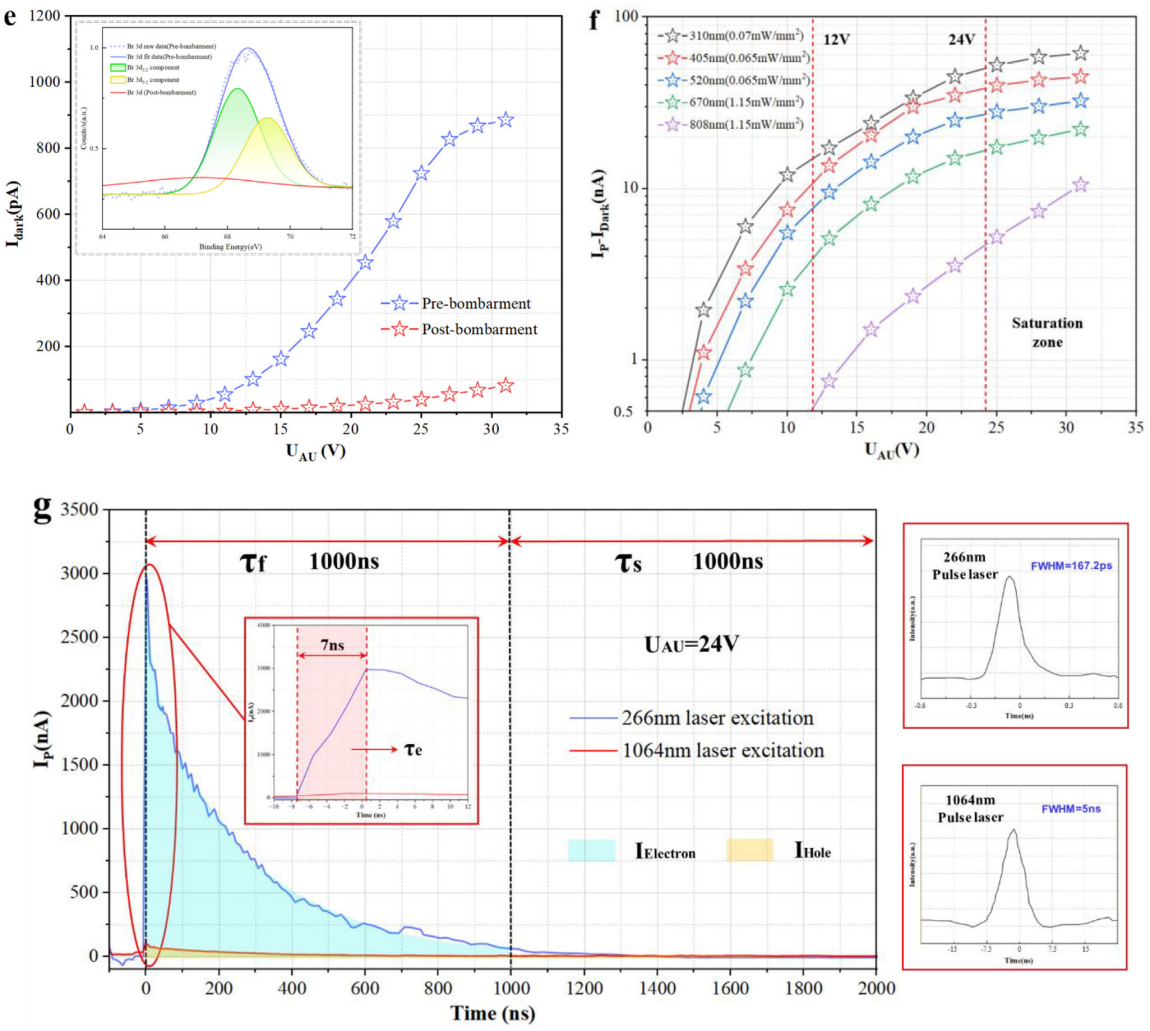

In contrast to the dark current, modeling the photocurrent density (J_p_) requires a dual integration over space and energy in Equation (3) because photo‐excited electrons can tunnel from the defect state to a spectrum of available states in the E_c_, each with a different probability weighted by the density of states D (E_c_’).

(3) $$\begin{array}{ccc} & & J_{p}\propto P_{tun}^{E_{t}->E_{c}}\propto \int_{0}^{w_{d}/w_{d}^{\prime }}\{\int_{E_{c}}^{\infty }exp\{-\frac{2\sqrt{2m_{t}^{*}}\lambda }{\hbar }\left[\left(E_{c}^{\prime }-E_{t}\right)-\Delta E_{k}\right.\\ & & \;-{\left.\Delta \phi_{bending}\left(x,U_{Au}\right)\right]}^{1/2}\}D\left(E_{c}^{\prime }\right)dE_{c}^{\prime }\}dx \end{array}$$

For 310, 405, or 520 nm weak illumination (≤0.1 mW/mm^2^) in Figure 1f, photon‐excited electrons trapped at defect states (E_t_) retain sufficient kinetic energy (ΔE_k_), enabling high tunneling probability from defect states to E_c_’ even at low U_Au_ (3∼6 V). The saturation of the 405 nm photoresponse above the U_Au_ of 24 V is a combined effect of a near‐unity tunneling probability from defect states to E_c_/E_c_’ and the saturation of electron drift velocity within the w_d_/w_d_’. For sub‐bandgap photons (670 nm, 808 nm) in Figure 1f, tunneling from defect states to E_c_ requires steeper band bending gradients and higher U_Au_. This is exemplified by the 808 nm response, where measurable photocurrent emerges only above a threshold bias of U_Au_ ≈12 V and subsequently increases gradually. Within the sub‐bandgap waveband, lowering photon energy corresponds to a contraction of the photocurrent‐generating tunneling zone. When sub‐bandgap photons extend into the NIR range, only electrode‐proximal Δϕ_bending_ (0, U_Au_) near Au contacts compensates the ΔE_k_ deficit.

The MSM transistor responds to NIR photons below 1064 nm, but the tunneling effect becomes undetectable exceeding 808 nm (1.54 eV), which quantitatively determines that Δϕ_bending_ (0, 12 V) is approaching the upper limit of EBR (Δϕ_max_ (0, U_Au_) ≈ E_g_−1.54 eV ≈ 0.88 eV). The Δϕ_max_ (0, U_Au_) directly predicts that defect‐mediated tunneling occurs within a length of three times the grain size (λ≈45 nm). In addition, this directly calibrates the ε_max_ ∼2×10^7^ V/m (Δϕ_max_ (0, 12 V)/λ) and w_d_ ∼1 µm (2λ×12 V/Δϕ_max_ (0,12 V)) via Equation (1). For NIR photons beyond 808 nm, whose energy is insufficient to provide any significant ΔE_k_, the observed photocurrent likely originates primarily from the hole‐dominated mechanism schematized in Figure 1d. To extract the dynamic fingerprint of defect‐mediated carrier transport in MSM transistors, we deconvolved electron‐ and hole‐dominated contributions via dual‐wavelength pulsed excitation (266 nm and 1064 nm). Once photo‐excited electrons successfully tunnel from defect states to the E_c_, the resulting free‐electron‐dominated current vastly exceeds the free‐hole contribution. The resulting free‐electron‐dominated photoconduction gain is expressed as G_e_ = τ_f_/τ_e_, where τ_e_ is the free electron transport time through the depletion region (w_d_/w_d_’) in Figure 1d, and τ_f_ is the average residence time of free electrons in the E_c_ (with its magnitude inversely proportional to the probability of an electron in E_c_ capture by defect levels). While the mobility of free electrons (µ_e_) nominally exceeds that of free holes (µ_h_), the orders‐of‐magnitude field difference across the depletion/non‐depletion zone dominates carrier dynamics. Within the high‐field depletion region (x≤w_d_), electrons rapidly accelerate to the saturation velocity under U_Au_ over 24 V. This reduces τ_e_ to values at least one order of magnitude smaller than the hole transit time τ_h_, as evidenced by the transient response in Figure 1g, which exhibits three distinct stages under 266 nm sub‐nanosecond pulse excitation. The first stage, a 7 ns rise time, is dominated by the electron transit time (τ_e_). The second stage is a 1000‐ns fall time. For a first‐order estimate, we approximate the defect‐capture process and attribute half of this decay duration to the average residence time (τ_f_ ≈ 500 ns). Thus, the free‐electron photoconductive gain can be calculated as G_e_ = τ_f_/τ_e_ ≈ 500 ns/7 ns ≈ 70‐fold. The third stage exhibits a slow decay exceeding 1000 ns, where the photocurrent eventually returns to the sub‐nA dark current level. This prolonged tail is predominantly attributed to free‐hole transport, based on its close resemblance to the hole‐dominated transient observed under 1064 nm pulsed excitation. The combined duration of this tail and the fall time (2τ_f_) corresponds to a defect relaxation time τ_r_ of ∼2 µs at room temperature. To deconvolve the carrier‐specific gains, we employed a geometric analysis by comparing the hole‐dominated region (reconstructed from the 1064 nm response) against the total photocurrent zone (from the 266 nm pulse). The area difference between these two regions provides a proportional estimate of the electron‐dominated current contribution. This analysis yields a hole‐dominated gain G_h_ of approximately fivefold, which is about an order of magnitude lower than the dominant electron gain G_e_ (≈ 70‐fold). In Figure 1g, when the 266 nm pulsed photon density far surpasses the bulk density of CsPbBr_3_ nanocrystals, the instantaneous electron‐dominated photocurrent approaches a saturated peak value (I_peak_) of 3 µA (reasonably neglecting the minor hole contribution G_h_), corresponding to ∼1 × 10^5^ free electrons per pulse (I_peak_×τ_e_/2). Operating in saturation mode (neglecting G_h_ contribution), the carrier number of defect‐mediated photocurrent is ∼1 × 10^5^. Given that these deep‐level defects induced by EB bombardment primarily localize at the interface or upper layers, the number of photo‐excited electrons per pulse (∼1 × 10^5^) corresponds to the number of active defects sampled within an effective detection area defined by the channel width (1 mm) and the w_d_ (∼1 µm). We reasonably estimate that the areal density of active defects reaches 2%–3% relative to that of CsPbBr_3_ nanocrystals at the interface [57], corresponding to a volumetric trap concentration (N_t_) likely exceeding 10^15^ cm^−3^ in the upper surface (1‐5 monolayers). These parameters (G_e_ ≈ 70, G_h_≈5, τ_r_≈2 µs, N_t_ > 10^15^ cm^−3^) collectively form the dynamic fingerprint of MSM transistors, extracted via deconvolution of dual‐wavelength transient responses.

Taken together, analogous to the charge‐transport role of ETL/HTL in photovoltaic devices, these functionally engineered deep‐level defects (via EB bombardment) serve as critical mediators for photo‐excited free‐electron transport in CsPbBr_3_ photocathode layers. This defect functionalization enables the conversion of non‐radiative energy (otherwise lost to photoluminescence) into a >70‐fold defect‐mediated photoconductive gain in the MSM transistor. The concomitant retention of strong native PL in these MSM transistors underpins their complementary optoelectronic functionality, permitting monolithic co‐integration of light emission and detection in single‐crystalline films to enable novel dual‐mode optical transceivers. Physically, the competition between transport/gain and PL originates from the law of energy conservation. The energy channeled into defect‐mediated transport and eventual photoemission is primarily harvested from non‐radiative pathways that would otherwise compete with radiative recombination (PL).

## 3 Defect‐Functionalized Interface Evolution Drives Photoemission

Figure 2a schematically illustrates our custom in situ system, which enables bi‐functional monitoring of both cathodoluminescence (CL) during EB bombardment and subsequent photoemission performance. Two solution‐processed CsPbBr_3_ films deposited on indium tin oxide (ITO) substrates function as photocathodes. The matched work functions between ITO (4.5–4.8 eV) [58] and the lightly doped p‐type CsPbBr_3_ (W_f_ ≈ 4.7±0.2 eV) [38] minimize the barrier for hole injection into the ITO, which is crucial for efficient hole extraction and preventing charge accumulation. Cross‐sectional thickness profiles of both photocathode films and X‐ray diffraction patterns of drop‐cast films from this solution are quantified in the inset of Figure 2a. Two deuterium lamps provide the photon flux for photoemission from both the Au and CsPbBr_3_ photocathodes. The system operates in two distinct modes. In the EB bombardment mode, a camera in Figure 2a monitors the CL of the CsPbBr_3_ films pumped by low‐energy EB (800 V) and high‐energy EB (2000 V). In the photoemission mode, one single picoammeter alternately quantifies the evolution of the photoemission current from both photocathodes. In addition, electron bombardment—our deliberate defect functionalization strategy—progressively aggregates and functionalizes deep‐level defects within the upper monolayers of CsPbBr_3_ films, irrespective of beam energy. Low‐energy beams (800 V) confine electron‐hole pair generation to this surface defect reservoir, maximizing non‐radiative recombination, while high‐energy beams (2000 V) penetrate deeply, generating pairs in the bulk far from surface defects. This spatial separation reduces non‐radiative losses, resulting in the enhanced CL efficiency observed in Figure 2b. For photoemission, these clustered deep‐level defects function as acceptor‐like states, forming localized EPABs. The high density of these positively charged centers (EPABs) effectively acts as a strong surface acceptor doping, transforming the upper surface into a heavily doped p‐type state. This transformation is directly verified by Kelvin probe force microscopy (KPFM) data in the inset of Figure 2b. The measured increase in work function (to 5.41 eV = W_Au_+0.31 eV and 5.47 eV = W_Au_+0.36 eV) is a direct consequence of the downward band bending induced by the positive charge associated with the EPABs. These results indicate a Fermi level shift toward the VBM, consistent with our earlier estimate of a high trap concentration (N_t_ ∼ 10^15^cm^−3^, or 2%). These data, reciprocal relationship experimentally verify the competitive energy dissipation mechanism between radiative recombination (PL or CL) and electron emission. Analogous to yet functionally distinct from the band bending at the Fermi‐level‐pinned Au/CsPbBr_3_ interface of MSM transistor, this transformation induces pronounced downward band bending at the CsPbBr_3_/vacuum interface. This bending serves as an activated layer of photoemission, effectively reducing the electron affinity (∆χ) and narrowing the energy offset between the interfacial vacuum energy level (E_vac’_) and the bulk E_c_. The formation of this band bending is analogous to the MSM case, with the built‐in potential (U_bi_) creating a bias‐free, built‐in field ɛ(x, U_bi_).

FIGURE 2Physical Origin of Defect‐mediated Photoemission in CsPbBr_3_ Films. (a) In situ EB bombardment/photoemission monitoring system with microchannel plates (MCPs). (b) Characteristic evolution of CsPbBr_3_ interfacial luminescence and photoemission during EB bombardment. (c) In situ comparison measurement based on MCPs. (d) Spectral QE distribution under CW excitation of CsPbBr_3_ photocathode from 200 to 450 nm. (e) Interfacial energy‐band features and electron escape dynamics of CsPbBr_3_ photocathode.

**Figure:**
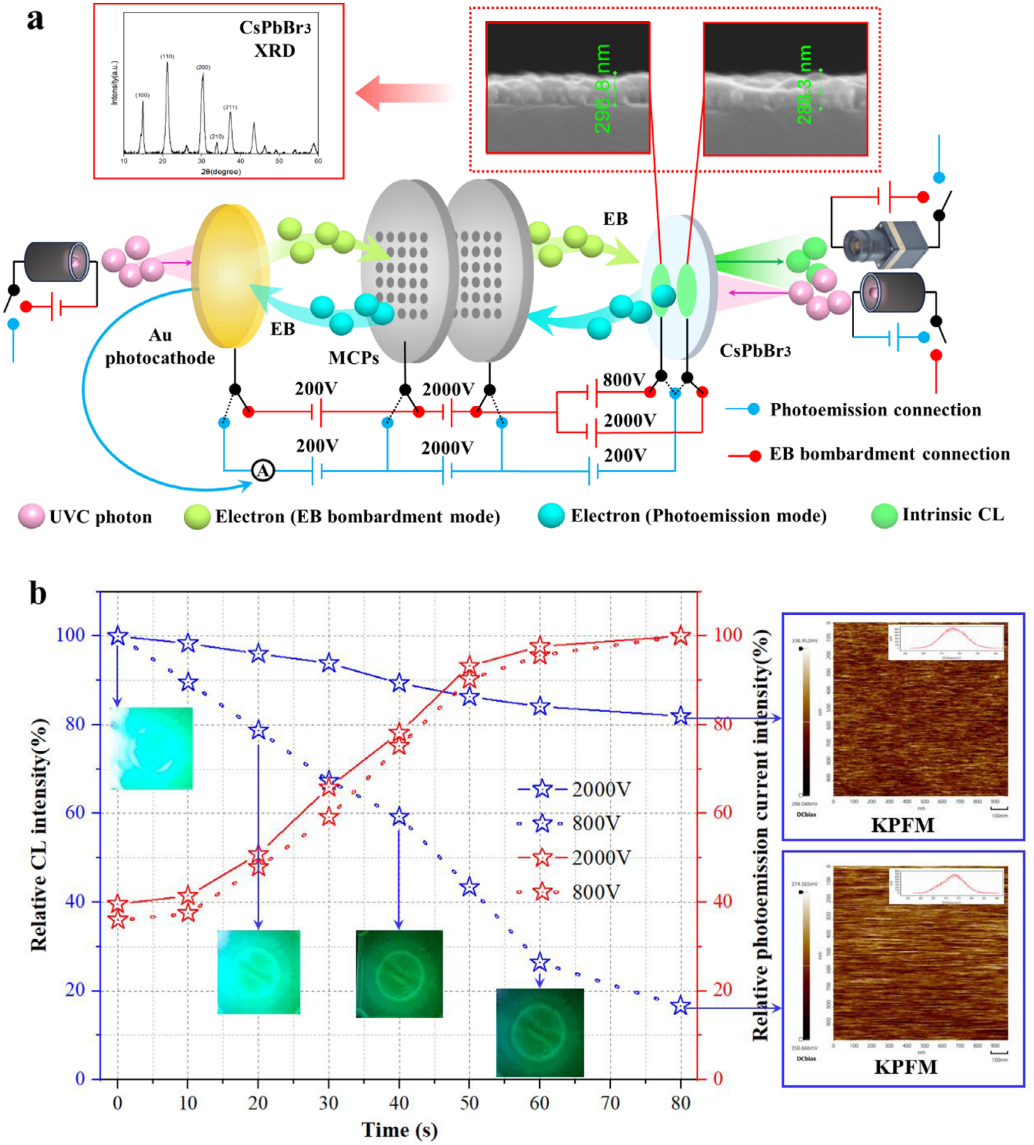

**Figure:**
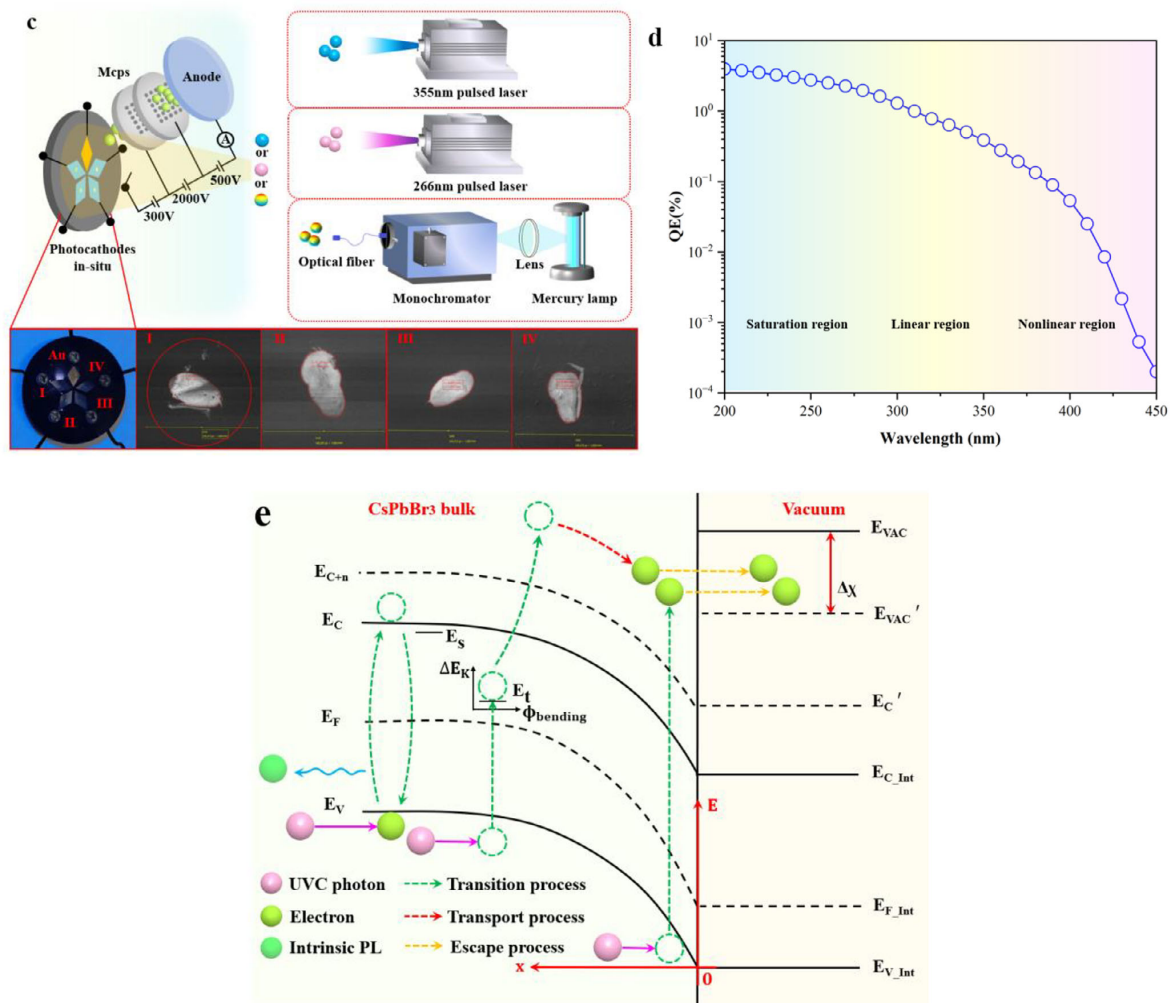

Crucially, the resulting interfacial band bending establishes a spatially varying built‐in field (ɛ(x, U_bi_)) across the film, which drives the directional drift and significantly enhances the transport efficiency of photo‐excited electrons. This built‐in field (ɛ(x, U_bi_)) synergistically promotes defect‐mediated tunneling for photoemission triggered by low‐energy photons, a mechanism directly analogous to that governed by ɛ(x, U_Au_) in Equation (1). Measuring these extremely low tunneling‐mediated photocurrents, particularly in the low‐QE visible range, poses a formidable challenge. However, conventional lock‐in amplification is ill‐suited for this task, as the stochastic nature of defect‐mediated tunneling generates a broad‐frequency noise spectrum that can overwhelm the modulated signal. To overcome this, we developed an in situ comparison measurement system based on MCPs as shown in Figure 2c, which provides the necessary gain and stable detection for low‐QE photoemission. This system estimates the absolute QE of the CsPbBr_3_ photocathodes by comparing their output to that of a reference photocathode. Specifically, it features a reference Au photocathode alongside the CsPbBr_3_ test spots. The absolute QE of the CsPbBr_3_ photocathode is calculated from the ratio of its MCP‐amplified signal to that of the Au reference, under identical illumination, scaled by the known QE of Au. To probe the interfacial band bending via the spectral QE distribution, we calibrated the system using a standard monochromatic light source (SML, 200–450 nm). The QE of the Au reference photocathode at 266 nm was first calibrated separately using lock‐in amplification, yielding a value of 0.5‱, consistent with literature values [59]. Using the calibrated MCP‐based measurement system in Figure 2c, the absolute spectral QE distribution of the CsPbBr_3_ photocathode was measured across the 200 nm to 450 nm range, as presented in Figure 2d. After normalizing for the absorptivity and valid area of the photocathodes, the QE at 266 nm for the EB‐activated CsPbBr_3_ film is calculated to be 1.14%, which is broadly consistent with values reported for Cs‐deposited CsPbBr_3_ photocathodes [39]. As shown in Figure 2c, this system categorized the QE distribution in Figure 2d into three distinct regions: a saturation region (≤280 nm), a linear region (280–355 nm), and a nonlinear region (>355 nm). In the saturation region (≤280 nm, hν > 4.43 eV), the photon energy is sufficient for photoemission even without significant assistance from Δϕ_bending_ (x, U_bi_), i.e., the effective vacuum barrier is overcome primarily by ΔE_k_. The observed QE saturation in this spectral region indicates a pronounced downward band bending of approximately 1.22 ± 0.1 eV (∆χ = IE‐4.43 eV) at the surface/vacuum interface, mediated by EPAB accumulation—a conclusion strongly consistent with the earlier KPFM data showing Fermi level shift and work function reduction in the insert of Figure 2b. In the linear region (280–355 nm, hv ≈ 4.43–3.49 eV), decreasing photon energy reduces the initial kinetic energy (ΔE_k_) of electrons excited into defect states. Consequently, the role of Δϕ_bending_ (x, U_bi_) in reducing the effective emission barrier becomes increasingly critical. Driven by the interfacial band bending gradient, the built‐in field ɛ (0, U_bi_) intensifies toward the EPABs on the surface, establishing a spatially graded enhancement of photoemission that peaks at the surface/vacuum interface. The depth of this effective photoemission layer shrinks as the photon energy decreases. Crucially, the spectral QE decay in the linear region of Figure 2d maps directly to contraction of the effective photoemission thickness, where Δϕ_bending_ (x, U_bi_) sufficiently lowers the energy barrier for photoemission. Notably, the energy span of the linear photoemission region (0.94 eV = 4.43–3.49 eV) in Figure 2d is comparable to the threshold energy for sub‐bandgap photoconductive gain (0.88 eV = 2.42–1.54 eV) in Figure 1f. This agreement suggests a similar physical origin: the maximum equivalent barrier reduction of Δϕ_bending_ (0, U_bi_) and Δϕ_bending_ (0, U_Au_) provided by the interfacial band bending is approximately 0.9 eV in both cases. Thus, the QE decay in the linear region of Figure 2d closely mirrors the photoresponse trend of the MSM transistor in Figure 1f, both governed by defect‐mediated tunneling modulated by band bending. This spectral QE distribution provides indirect insight into the CsPbBr_3_ photocathode film's interfacial energy‐band features and elucidates the microscopic pathway of photo‐excited electrons during linear photoemission, from defect‐mediated transport to their eventual escape across the interface in Figure 2e. In summary, the evolution of Figure 2b traces the dynamic process of EB‐generated defects concurrently inducing energy band bending and vacuum barrier reduction at the photocathode interface while mediating exciton dissociation/transport in Figure 1d. Functionally, this EB bombardment constitutes a defect‐functionalized physical activation approach for CsPbBr_3_ photocathode films, distinct from traditional chemical techniques. Most significantly, the QE within the visible range exhibits a pronounced non‐linear enhancement, providing a clear signature of nonlinear photoemission.

## 4 Defect‐Mediated Nonlinear Photoemission Enables Transient QE Multiplication

The excess kinetic energy (ΔE_k_) of electrons in defect states, as defined in Equation (3), is the critical parameter governing nonlinear photoemission. It originates from two sources: the energy excess from the primary photoexcitation event (hν—E_t_), and crucially, from secondary photon‐driven excitation. The secondary excitation process provides the physical pathway for a significant boost in ΔE_k_ under pulsed excitation. The probability P_ΔE_ (Φ) that a trapped electron undergoes secondary excitation during a pulse depends on the competition between the photoexcitation rate and the defect relaxation rate in Equation (4).

(4) $$P_{\Delta E}\left(\Phi \right)=\left(\frac{\sigma_{d}\Phi }{\sigma_{d}\Phi +{\tau_{r}}^{-1}}\right)\times \left\{1-exp\left[-\left(\sigma_{d}\Phi +\frac{1}{\tau_{r}}\right)\Delta t\right]\right\}$$

The microsecond‐scale τ_r_ allows a finite, albeit very low, probability for secondary excitation under low‐flux CW illumination (governing the nonlinear region in Figure 2d). This probability, however, becomes significant under pulsed excitation. Secondary photon‐driven excitation of defect‐bound electrons enhances ΔE_k_ while concurrently lowering the effective vacuum barrier via Δϕ_bending_ (x, U_bi_), thereby synergistically extending the spectral response of CsPbBr_3_ photocathodes into the visible range. As in the linear region, decreasing photon energy in the nonlinear region further reduces the effective photoemission depth, as a steeper band bending gradient (Δϕ_bending_ (x, U_bi_)) is required to compensate for the lower ΔE_k_. As a first‐order model neglecting the competing PL channel, the local QE distribution of CsPbBr_3_ photocathode can be qualitatively described as Equation (5).

(5)

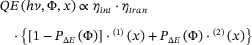

where *η*_int_ is the interface transmission efficiency (0 to 1), η_tran_ is the free electrons transport probability in the E_c_. For our 300‐nm films, we calculate η_tran_ to exceed 98%, based on the relationship η_tran_ = (1‐ (τ_e_/2τ_f_) × (300 nm/w_d_)) and the values of τ_e_ and τ_f_ from Figure 1g. The kinetic energy of an electron following primary excitation into a defect state is ΔE_k_ = max (0, hν−E_t_). The terms Π ^(1)^ and Π ^(2)^ represent the composite probabilities for electron emission via the two distinct excitation pathways:


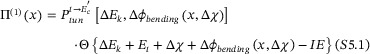




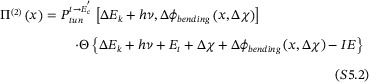

where Θ(x) represents the Heaviside step function for Photoemission. Under low‐flux CW excitation, the secondary excitation probability P_ΔE_ (Φ) approaches zero. Theoretically, a higher intensity of CW illumination would enhance the probability of secondary excitation occurring within the τ_r_, thereby boosting photoemission in the nonlinear region of Figure 2d. However, high‐intensity CW excitation severely threatens thermal stability [60, 61], as demonstrated in Figure 3a. Specifically, 266‐nm CW illumination at 800 mW/cm^2^ raises the local temperature in 300‐nm CsPbBr_3_ films above 140°C in Figure 3a, approaching its decomposition temperature [62]. Critically, the low peak photon density of CW illumination fails to effectively excite deep‐level defects within the microsecond‐scale τ_r_. This ineffective defect excitation directly contributes to the low QE in the nonlinear region of Figure 2d. Conversely, 266‐nm pulsed excitation maintains thermal stability (<10°C rise at equivalent average power) while delivering peak photon densities orders of magnitude beyond CW illumination, enabling transient QE multiplication of CsPbBr_3_ photocathode. Figure 3b illustrates the microscopic mechanisms of defect‐mediated secondary excitation under CW versus pulsed modes. In Figure 3a, the minimal transient heating under pulsed excitation provides indirect evidence for this energy partitioning: it suggests that a larger fraction of the energy absorbed from the pulse is channeled into electron emission rather than lattice heating. Using the in situ system from Figures 2c and 3c provide experimental validation of the mechanisms proposed in Figure 3b, demonstrating QE multiplication evolution of 266‐nm (saturation) and 355‐nm (linear/nonlinear) pulsed excitation. Here, the gain of QE multiplication is defined as 20lg (QE_pulsed_/QE_cw_) at fixed average incident optical power, and its photoemission can be depicted as Equation (6).

(6) $$Q_{total}\left(h\nu ,\Phi \right)\propto f_{t}\cdot d_{eff}\cdot \int N_{t}\left(x\right)\cdot QE\left(h\nu ,\Phi ,x\right)dx\;$$

where f_t_ is the defect occupation fraction, d_eff_ is the effective photoemission area, N_t_ (x) is the bulk density of deep‐level defects at depth x, whose experimental value may exceed 10^15^ cm^−3^ in the upper layers. Under 266 nm pulsed excitation, the QE rises from 1.14% (CW) to 8.4%, indicating that secondary excitation significantly increases the participation of defect‐bound electrons in the photoemission process. The QE of 8.4% approaches the theoretical maximum contribution expected from non‐radiative pathways, estimated as (100%—PLQY) [63, 64]. The absolute gain is constrained by the relatively high baseline CW QE (>1%) in the saturation region. Moreover, 355‐nm pulsed excitation provides sufficient energy (3.49 eV + E_t_>IE‐Δχ) through secondary excitation to dominate nonlinear photoemission, rendering it independent of spatial band bending variations (Δϕ_bending_ (x, U_bi_)). As demonstrated in Figure 3c, 355‐nm pulsed excitation achieves a far greater relative QE gain compared to 266‐nm pulsed excitation, due to its much lower CW baseline. The convergence of the QE values at both wavelengths under pulsed excitation highlights the dominance of nonlinear photoemission, with its upper limit set by the non‐radiative recombination efficiency. Consequently, the QE multiplication in Figure 3c stems solely from high‐density pulses redirecting defect‐driven non‐radiative energy losses from thermal dissipation to electron emission. Suppressing PLQY while preserving critical band structure characteristics constitutes the optimal strategy for further enhancing transient QE. Therefore, we incorporated Au nanocrystals (Au@NCs) into the CsPbBr_3_ precursor solution to intentionally introduce additional scattering centers and defect states, with the dual purpose of modifying the defect landscape and suppressing the PLQY. These incorporated scattering centers increase the effective photon path length throughout the CsPbBr_3_ film, leading to an effectively larger excitation cross‐section (σ_d’_) for deep‐level defects. The incorporated Au@NCs induce lattice strain that increases the density of shallow traps (E_s_), promoting non‐radiative recombination and thereby suppressing PLQY. We hypothesize that the increased shallow‐trap density channels more charge carriers through the deep‐level recombination centers, potentially increasing their f_t_. Low‐temperature PL spectra in Figure 3d shows that increasing Au@NCs concentration or proportion enhances the shallow‐defect emission peaks relative to the band‐edge peak, supporting the claim of an increased E_s_ density. The in situ “transmission‐mode” EDS mapping confirms the incorporation and distribution of Au.

FIGURE 3QE multiplication measurement and QE gain modulation of nonlinear photoemission. (a) Thermal stability comparison in a 300‐nm‐thick CsPbBr_3_ film under CW vs pulsed excitation. (b) Microscopic physical processes of defect‐mediated secondary excitation under CW versus pulsed modes. (c) QE evolution of CsPbBr_3_ photocathode under pulsed excitation. (d) Low‐temperature PL spectrum and in situ “transmission‐mode” EDS mapping of hybrid CsPbBr_3_ photocathode. (e) Au@NCs proportion‐dependent photoemission and PL intensity evolution of CsPbBr_3_ film with transmission electron microscopy (TEM) characterization. (f) In situ observation of nonlinear photoemission via self‐pumping by bandgap PL.

**Figure:**
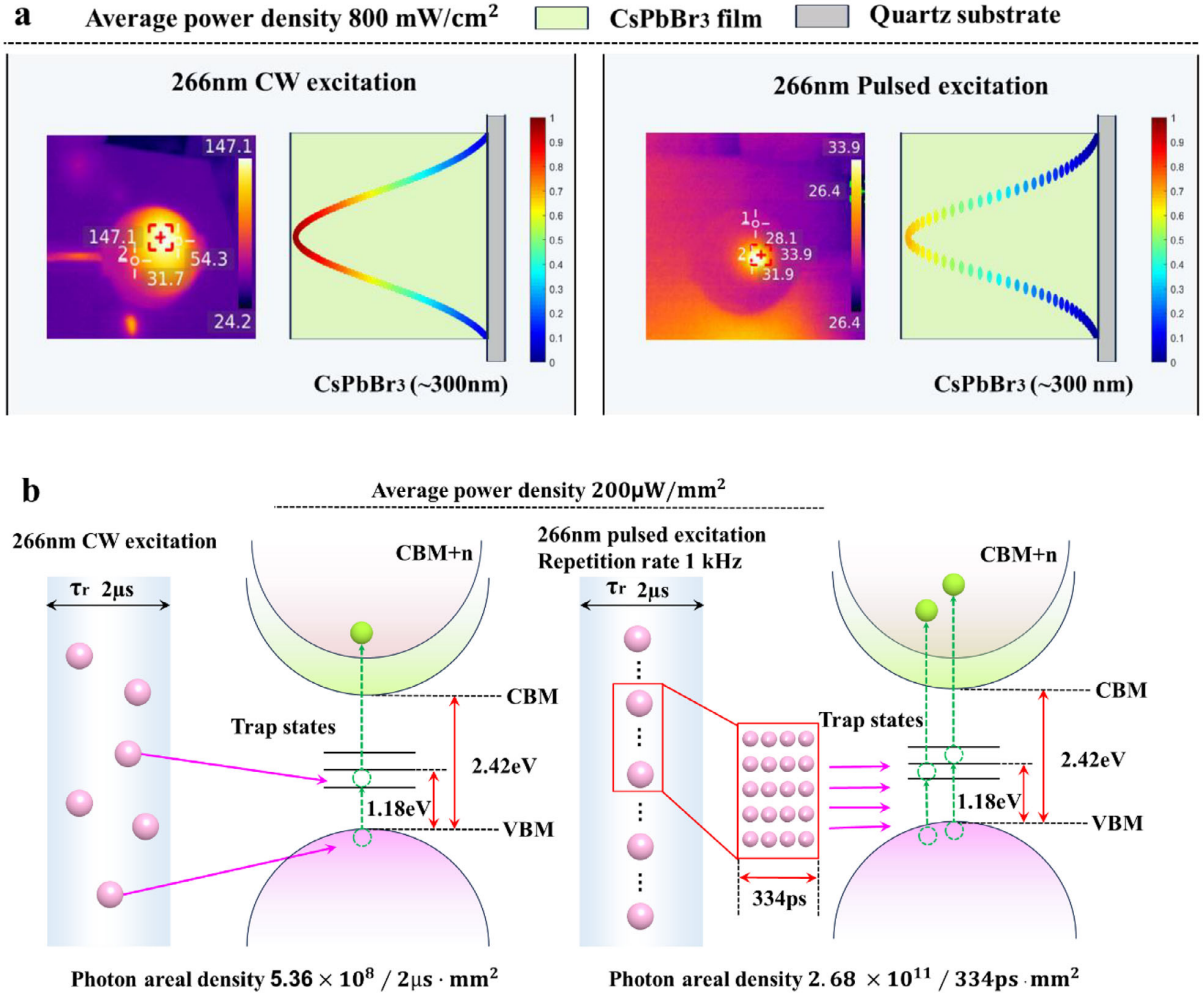

**Figure:**
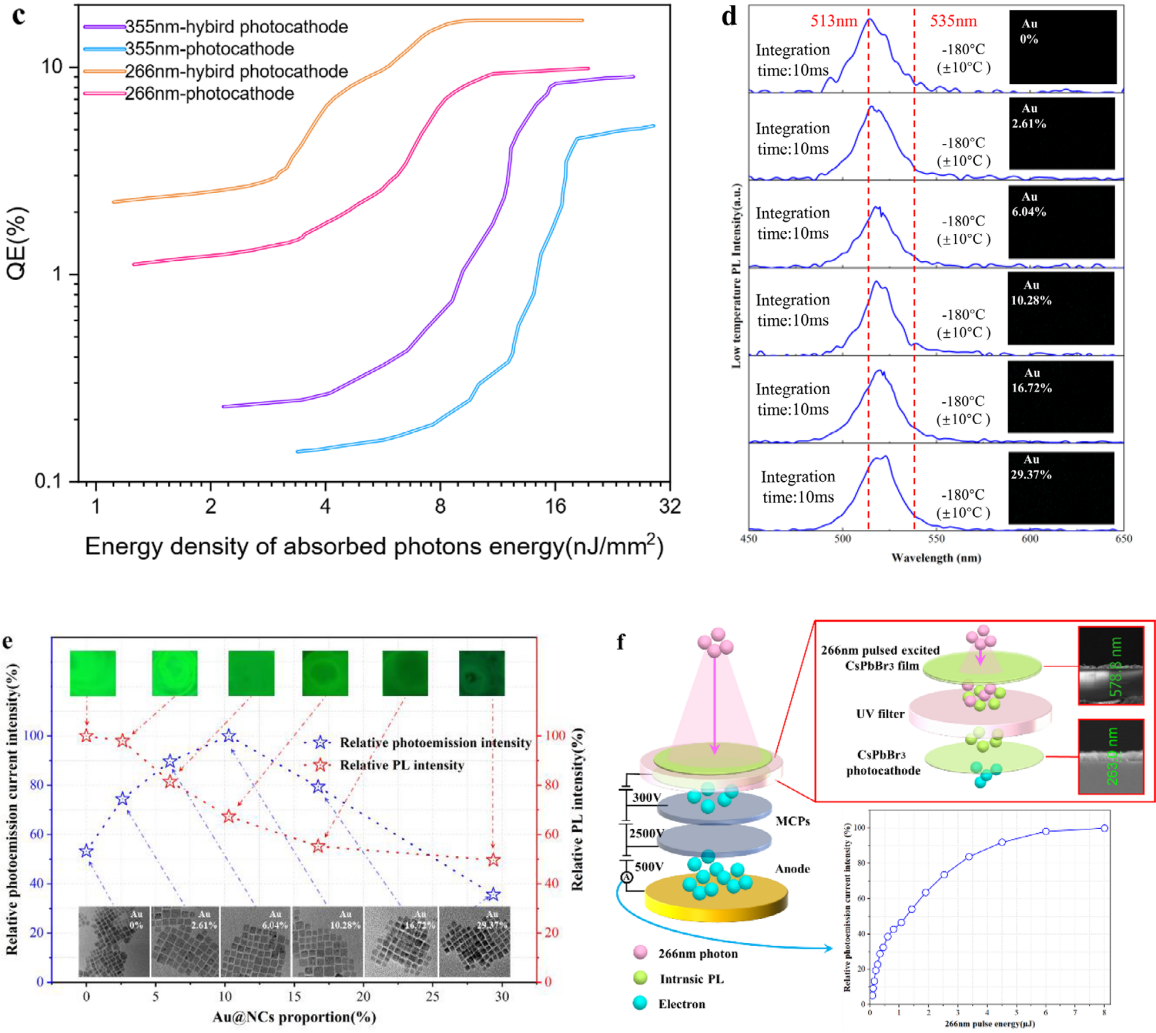

Undeniably, quantitative control of defect distribution remains challenging, however, as excessive Au@NCs incorporation triggers nanoparticle clustering [65], non‐linearly degrading photoemission efficiency (e.g., η_tran_, d_eff_). In Figure 3e, the proportion‐dependent photoemission evolution reveals near‐doubling of CW photoemission intensity at UV wavelengths under optimal doping. Corresponding TEM insets of Figure 3e demonstrate the direct correlation between photoemissive performance and Au@NCs hybridization concentration. The right axis of Figure 3e further presents the evolution of relative PL intensity in the physically hybrid CsPbBr_3_ films, where identified E_s_ effectively suppress radiative recombination. Using the optimally doped photocathode from Figure 3e, we measured the pulsed QE at 266 and 355 nm. The resulting QE evolution curves for these hybrid films are added to Figure 3c for comparison. Under 266‐nm pulsed excitation, the hybrid CsPbBr_3_ photocathode achieves a peak transient QE of 16.8%. Although the 16.8% transient QE remains below the >20% linear QE of NEA photocathodes [15], the >15‐fold multiplication under 266 nm pulsed excitation in Figure 3c endows this class of nonlinear photocathodes with a fundamentally new device function. This is exemplified by the 34 dB gain at 355 nm, which functionally realizes a photoemissive comparator, capable of compressing optical pulses into ultrashort electron pulses in space and time. To provide direct evidence of the nonlinear gain, we designed an in situ self‐pumping experiment in Figure 3f: the bandgap PL pulse from one CsPbBr_3_ film serves as the excitation source for a second, identical photocathode. This scheme bypasses external lasers and directly demonstrates giant nonlinear photoemissive multiplication, representing a transition from undetectable CW emission to readily measurable nonlinear gain. As a control experiment, 532‐nm pulsed excitation (2.32 eV) fails to elicit detectable nonlinear photoemission because the total energy (2.32 eV+E_t_) is estimated to be below the effective ionization energy (IE‐Δχ‐Δϕ_bending_ (0, U_bi_)). This spectral selectivity confirms the existence of a nonlinear threshold. We estimate the threshold photon energy to be approximately 2.5‐2.6 eV, above which the condition (hν + E_t_ > IE‐Δχ‐Δϕ_bending_ (0, U_bi_)) is satisfied. The transient QE multiplication factor near the bandgap could not be directly quantified due to the absence of reliable near‐bandgap CW QE data and challenges in accurately measuring the transient PL intensity used as the excitation source in Figure 3f.

## 5 Conclusion

Deep‐level defects in perovskites are conventionally viewed as detrimental traps that quench PL. In this work, we repurpose these defects to harness non‐radiative losses, transforming them into functional mediators that enable nonlinear photoemission via defect‐mediated tunneling. This work establishes defect‐mediated tunneling as a physical mechanism that repurposes non‐radiative losses into nonlinear photoemission in CsPbBr_3_. This mechanism enables device functionalities in two distinct domains, as summarized below.

**In solid‐state photonics**, the defect‐mediated tunneling mechanism enables Au/CsPbBr_3_/Au MSM transistors to achieve photoconductive gains exceeding 70‐fold while maintaining sub‐nA dark current. This gain is obtained without dedicated charge transport layers, offering an alternative design approach for perovskite photodetectors. The combination of high gain and low dark current is of interest for all‐solid‐state photodetection. Notably, defect‐mediated tunneling fundamentally exploits non‐radiative energy losses in CsPbBr_3_ PL to generate photoconductive gain—turning “wasted energy” into a design resource for solid‐state device performance enhancement. Additionally, the same films retain their native luminescence, suggesting potential for monolithic co‐integration of emission and detection in single‐crystalline architectures. Furthermore, the tunneling mechanism extends the spectral response to 808 nm (a >0.88 eV extension beyond the bandgap) and exhibits voltage‐tunable detection thresholds, providing wavelength‐discriminating capability without external optical filters.

**Extending this framework to vacuum photonics**, we demonstrate that EB bombardment activates the CsPbBr_3_ photocathode interface via defect‐mediated band bending. This physical activation method is distinct from conventional chemical activation and operates under moderate vacuum conditions. On this basis, we demonstrate nonlinear photoemission with transient QE multiplication. Under 266 nm pulsed excitation, the QE reaches 16.8% and a 34 dB gain is achieved at 355 nm. This nonlinear response functionally demonstrates a photoemissive comparator, with potential application in spatiotemporal optical pulse compression.

## 6 Experimental Section/Methods

### 6.1 CsPbBr_3_ Preparation and Characterization

CsPbBr_3_ quantum dots were synthesized at 180°C under an inert atmosphere using hot‐injection with cesium oleate and lead bromide as precursors, followed by a ligand exchange reaction for 60 s. The resulting perovskite nanocrystals were purified by centrifugation for 10 min, yielding a hexane dispersion with a concentration of 10 mg/mL that exhibits green emission peaking at 513 nm. X‐ray diffraction patterns of drop‐cast films from this perovskite solution are presented in the inset of Figure 2a.

### 6.2 Hybridization of CsPbBr_3_ Solution With Au Nanocrystals

Commercially sourced Au@NCs with a diameter of 5 nm and dodecanethiol as ligands, were purchased from Taobao (China) as a dispersion in hexane at a concentration of 0.1 mg/mL (customized product, no batch number). The Au@NCs were physically blended into the CsPbBr_3_ precursor solution (hexane) at controlled concentrations without further modification. To ensure uniform dispersion, the hybrid solution was heated at 60°C for 30 min with agitation on an orbital shaker at 200 rpm. The dispersion was then subjected to magnetic stirring at 600 rpm for 10 min. Films were deposited on ITO via spin‐coating using a two‐step program: 900 rpm for 8 s with an acceleration of 500 rpm/s, followed by 3500 rpm for 15 s with an acceleration of 1000 rpm/s.

### 6.3 Measurement of In Situ “Transmission‐Mode” EDS

The distinctive feature of our in situ “transmission‐mode” EDS technique is that the SEM's anode is positioned to collect electrons that are transmitted through the sample, enabling direct compositional analysis of the same micro‐area undergoing electron bombardment. Two distinct experimental configurations were utilized in this work. In one, applied to Figure 1b, the interdigitated Au electrodes of the MSM transistor themselves functioned as the anode. This setup allowed the focused incident electron beam to bombard the device under an applied electrical bias (U_Au_), encompassing both the Au electrodes and the semiconductor channel. Consequently, this approach not only enables the simultaneous monitoring of electron bombardment‐induced interfacial composition changes and the resultant evolution of photoelectronic properties, but also provides a direct correlation between physical transformation and device function. In the other, used for Figure 3d, the ITO substrate served as the anode. This design was adopted to avoid the potential physical and chemical damage to the delicate CsPbBr_3_ film that could result from fabricating additional dedicated anode contacts, thereby ensuring the integrity of the film and its interfaces for analysis.

### 6.4 Luminescence Power Calibration of the SML Source

The SML source integrates a 2000 W high‐pressure mercury lamp with a monochromator (5 nm spectral bandwidth). To mitigate lamp‐specific spectral variance and temperature‐dependent line intensity modulation, spectral irradiance is recalibrated prior to each measurement campaign using a UV‐Vis spectrometer. Precision optical attenuators positioned at the lamp output port regulate photon flux at the fiber‐optic terminus to 200 µW—5 mW with ±3% temporal stability, ensuring incident power accuracy of ±5% for absolute QE characterization in Figure 2d.

### 6.5 Au Photocathode Preparation and Characterization

A 100 nm thick Au film was deposited onto fused quartz substrates by electron‐beam evaporation at a rate of 0.5 nm/s under a base pressure of <5 × 10^−7^ Torr. We characterized the absolute QE of this Au reference at 266 nm using the calibrated SML source. The SML output at 266 nm was attenuated to an irradiance of 20 µW/cm^2^ over a spot size of 20 mm^2^ and irradiated onto the Au photocathode under a vacuum of <10^−6^ Torr. Photocurrent signals were measured via lock‐in amplification at 400 Hz modulation frequency. QE was calculated from the photoelectron yield relative to incident photons. This yielded a reproducible QE at 266 nm, consistent with established values for metallic photocathodes. The calibrated QE enabled direct performance benchmarking of CsPbBr_3_ photocathodes in Figures 2 and 3.

### 6.6 Measurement of Temperature‐Dependent PL Spectra

Optical characterization employed a visible spectrometer (380–780 nm) and an infrared spectrometer (900–2500 nm). The low‐temperature spectra presented in the insets of Figures 1 and 3 were acquired with these instruments. Photoluminescence was excited by a 405 nm semiconductor laser at a power density of 100 mW/cm^2^. The sample was cooled by liquid nitrogen.

### 6.7 Calibration of KPFM Against an Au Standard

The KPFM probe was calibrated using a standard Au film with a nominal work function of 5.1 eV. A uniform region was selected for potential mapping, yielding an average contact potential difference (CPD) of 51.861 mV. The standard deviation of the CPD across this region was ±5 mV, which corresponds to an uncertainty in the derived work function values of approximately ±0.05 eV.

### 6.8 Vacuum‐Limited Stability of CsPbBr_3_ Photocathodes in In Situ Systems

The three distinct in situ systems integrated with MCPs were used across Figures 2 and 3 to evaluate the nonlinear photoelectric performance of CsPbBr_3_ films. Although conventional photocathodes demand ultrahigh vacuum (UHV), our CsPbBr_3_ photocathodes omitted cesium deposition and functioned reliably at 10^−6^ torr throughout all experiments. Crucially, CsPbBr_3_ photocathode films exhibit exceptional low‐vacuum tolerance, showing almost no QE degradation even after prolonged storage at several torr. To accelerate ion feedback‐induced degradation, we conducted continuous 150‐h operational tests at 10^−5^ torr. The QE progressively declined to 50% of the activated state after 50 h and further decreased to 30% of peak performance after 150 h. Notably, as demonstrated in Figure 2b, compromised QE was fully restored via EB‐bombardment reactivation—validating the self‐recovery mechanism in Figure 2a. The recovery process was highly reproducible, with the rejuvenation kinetics directly correlated to the surface defect density and distribution. This reversible recovery enables sustainable operation at moderate vacuum (10^−6^ torr), overcoming traditional UHV constraints while maintaining stability in compact in situ systems.

### 6.9 VBM and E_f_ Measurement of CsPbBr_3_ Film via UPS

The valence band spectra of CsPbBr_3_ films are measured with a monochromatic He lamp source (21.2 eV) and a VG Scienta R4000 analyzer. A sample bias of −5 V was applied to observe the secondary electron cutoff (SEC). The work function (E_f_) and VBM can be determined by the difference between the photon energy and the binding energy of the secondary cutoff edge.

### 6.10 Reproducibility of QE Multiplication and Nonlinear Photoemission

As illustrated in Figure 2c, the in situ photocathode assembly comprises a five‐electrode array, integrating one reference Au photocathode with four testing photocathode spots. The QE multiplication data presented in Figure 3c were derived from the averaged photocurrent across multiple testing photocathode spots. Furthermore, the transient QE value and its corresponding QE gain, obtained from five sequential QE multiplication measurements, demonstrated excellent reproducibility with a variation of less than 10%.

### 6.11 EB Bombardment Conditions for Defect Functionalization

The acceleration voltage of EB bombardment was varied between 500 and 5000 V, with typical operating conditions of 2000 V for high‐energy bombardment and 800 V for low‐energy bombardment (as used in Figure 2b). The beam current density was maintained at approximately 50–200 nA/mm^2^. Bombardment duration ranged from 30 to 200 s, depending on the desired degree of defect functionalization. All bombardments were performed at room temperature under a vacuum of <10^−5 ^Torr. Acceleration voltages exceeding 6000 V, particularly under continuous bombardment, caused visible damage to the CsPbBr_3_ film and were therefore avoided.

### 6.12 Effect of Storage Conditions on Defect Evolution and Device Performance

In principle, storage conditions can influence both the composition and defect states of CsPbBr_3_ films. For example, an MSM transistor identical to that shown in Figure 1a, if exposed to dry ambient atmosphere for several weeks, can develop weak photoconductive behavior qualitatively similar to Figure 1e,f—though with far inferior performance and, critically, without spatial controllability. Conversely, when such devices are stored under vacuum for extended periods, defect generation appears to be significantly passivated. At the very least, from the perspective of MSM transistor photoconductivity and CsPbBr3 photocathode performance, we cannot detect any measurable changes in defect states induced by vacuum storage alone. The stark contrast between the rapid, controllable effects of EB bombardment and the slow, uncontrolled evolution under passive storage conditions further reinforces the central role of EB‐induced defect functionalization in our work.

## Funding

(Foundation Project of Jilin Province (No.20240101300JC); National Natural Science Foundation of China (U2141239)).

## Conflicts of Interest

The authors declare no conflicts of interest.

## Data Availability

The data that support the findings of this study are available from the corresponding author upon reasonable request.
